# Comparison of Protein- or Amino Acid-Based Supplements in the Rehabilitation of Men with Severe Obesity: A Randomized Controlled Pilot Study

**DOI:** 10.3390/jcm12134257

**Published:** 2023-06-25

**Authors:** Amelia Brunani, Raffaella Cancello, Michele Gobbi, Elisa Lucchetti, Giulia Di Guglielmo, Sabrina Maestrini, Stefania Cattaldo, Paolo Piterà, Chiara Ruocco, Alessandra Milesi, Alessandra Valerio, Paolo Capodaglio, Enzo Nisoli

**Affiliations:** 1 IRCCS, Istituto Auxologico Italiano, Ospedale San Giuseppe, Piancavallo, 28921 Verbania, Italy; m.gobbi@auxologico.it (M.G.); e.lucchetti@auxologico.it (E.L.); g.diguglielmo@auxologico.it (G.D.G.); s.maestrini@auxologico.it (S.M.); s.cattaldo@auxologico.it (S.C.); p.pitera@auxologico.it (P.P.); a.milesi@auxologico.it (A.M.);; 2 Obesity Unit, Department of Endocrine and Metabolic Diseases, Laboratory of Nutrition and Obesity Research, IRCCS Istituto Auxologico Italiano, 20100 Milan, Italy; r.cancello@auxologico.it; 3 Center for Study and Research on Obesity, Department of Biomedical Technology and Translational Medicine, University of Milan, 20100 Milan, Italy; chiararuocco@gmail.com (C.R.);; 4 Department of Molecular and Translational Medicine, University of Brescia, 25121 Brescia, Italy; 5 Department of Surgical Sciences, Physical and Rehabilitation Medicine, University of Torino, 10121 Torino, Italy

**Keywords:** obesity, amino acids, proteins, essential amino acids, tricarboxylic acids, supplementation, diet, weight loss

## Abstract

Background: Weight loss is associated with a reduction in all body compartments, including muscle mass (MM), and this effect produces a decrease in function and muscle strength. Our objective was to assess the impact of protein or amino acid supplements on MM loss in middle-aged men (age < 65 years) with severe obesity (BMI > 35 kg/m^2^) during weight loss. Materials and Methods: We conducted a single-site randomized controlled trial (Clinicaltrials.gov NCT05143398) with 40 in-patient male subjects with severe obesity. Participants underwent an intervention program consisting of a low-calorie balanced diet and structured physical activity. They were randomly assigned to 4-week treatment groups: (1) control (CTR, N = 10), (2) protein (P, N = 10), (3) branched-chain amino acid (BCAA, N = 10), and (4) essential amino acid mixture with tricarboxylic acid cycle intermediates (PD-E07, N = 10) supplementation. Results: Following 4 weeks of intervention, all groups showed similar reductions in body weight compared to baseline. When examining the delta values, a notable increase in muscle mass (MM) was observed in the PD-E07 intervention group [MM (kg): 2.84 ± 3.57; MM (%): 3.63 ± 3.14], in contrast to the CTR group [MM (kg): −2.46 ± 3.04; MM (%): −0.47 ± 2.28], with a statistical significance of *p* = 0.045 and *p* = 0.023, respectively. However, the MM values for the P group [MM (kg): −2.75 ± 5.98, *p* = 0.734; MM (%): −0.44 ± 4.02, *p* = 0.990] and the BCAA group [MM (kg): −1 ± 3.3, *p* = 0.734; MM (%): 0.34 ± 2.85, *p* = 0.956] did not exhibit a statistically significant difference when compared to the CTR group. Conclusions: Amino acid-based supplements may effectively mitigate the loss of MM typically observed during weight reduction. Further validation through large-scale studies is necessary.

## 1. Introduction

The current goal in treating obesity is to achieve a 5–10% reduction in body weight, which can be accomplished through lifestyle interventions such as diet, exercise, and available drugs [1,2]. According to the latest reports published by the American College of Sports Medicine [3,4], physical exercise is widely recognized and a fundamental component of weight loss programs, both at the European and global levels [3,4]. Regular physical activity in weight loss interventions helps improve cardiovascular health, increases metabolic rate, enhances muscle strength and endurance, and contributes to overall well-being [4]. However, weight loss, especially without regular exercise, can lead to unintended loss of fat-free mass (FFM), particularly muscle mass (MM), in addition to fat mass (FM) loss, particularly among older individuals, thereby increasing the risk of developing sarcopenia [5,6]. For instance, a 24-week calorie restriction without resistance training resulted in the loss of FFM and MM and a decline in functional strength [7]. Notably, FFM accounts for 20–30% of total weight loss, which is more pronounced in males than females, potentially adversely affecting patients with obesity [7]. Moreover, the decline in FFM and MM is associated with an increased risk of falls and physical disabilities [8]. The skeletal muscle plays a crucial role in maintaining body homeostasis, accounting for over 75% of all insulin-mediated glucose disposal [9]. Disruptions in skeletal muscle function can negatively impact metabolic processes [9]; in contrast, muscle strength has a positive correlation with increased insulin sensitivity and a negative association with cardiovascular (CVD) risk and mortality [10,11,12,13]. Additionally, the loss of muscle mass (MM) is linked to intramuscular lipid infiltration, a characteristic commonly found in skeletal muscle in obese patients [8]. However, during aerobic exercise, lipid oxidation contributes to MM increase and decreases lipid depots [14].

The debate continues regarding the optimal macronutrient composition of a diet that promotes sufficient energy deficit, reduces body fat mass, and maintains or improves lean body (muscular) mass while preserving functional performance [10]. The currently available data show that weight-loss therapy, including a hypocaloric diet (with protein intake ≤1.0 g for Kg of ideal body weight) and regular physical activity, should be promoted to maintain muscle mass and improve muscle strength and physical function in patients with obesity [15,16]. The primary metabolic process for preserving muscle mass in the body is protein turnover, which involves a balance between muscle protein synthesis and degradation [11]. The essential amino acids (EAAs), particularly leucine, are crucial in protein synthesis stimulation [12]. Dietary amino acids with exercise training induce a synergic effect on protein turnover, enhancing skeletal muscle mass [17]. Nonetheless, when high protein intake (e.g., >1.5 g/kg body weight/day beyond recommended daily allowances for protein) is combined with dietary restriction, with or without exercise training, there is considerable variability in the outcomes concerning body weight modulation, body composition, the maintenance or increase in fat-free mass (FFM), and the management of metabolic indexes in patients that are overweight or have been diagnosed with obesity [10,17,18,19,20].

The supplementation with branched-chain amino acids (BCAA) formulas was recently proposed as a promising approach to managing elderly obese or sarcopenic patients [12,16,21,22,23]. These BCAA-based supplements have been shown to optimize muscle protein synthesis during an energy deficit, counteracting protein disarrangement and preserving energy homeostasis in acute and chronic hypercatabolic conditions without impacting on renal function [24,25]. In a previous study, we demonstrated the effectiveness of a new BCAA-enriched mixture (BCAAem) in improving physical and cognitive performance in malnourished older adults [21]. Additionally, we observed a strong correlation between clinical improvement and the bioenergetics of peripheral blood mononuclear cells [21]. Our findings have demonstrated that the BCAAem mixtures have remarkable effects on health and longevity in aged mice. Specifically, these mixtures have been shown to preserve mitochondrial energy efficiency and enhance the defense system against radical oxygen species (ROS) [26]. In a recent study, we developed a protein-restricted diet enriched with free essential amino acids (EAAs). This diet has proven effective in preventing and reversing obesity and restoring dysregulated glucose homeostasis in various mouse models, ultimately extending their healthy lifespan [27]. Furthermore, we have introduced two novel amino acid formulas, namely α5 and PD-E07, which exhibit well-balanced stoichiometric ratios of EAAs and BCAAs, as well as tricarboxylic acid cycle (TCA) precursors and cofactors such as citric, succinic, and malic acid. These formulas have been specifically designed to optimize mitochondrial bioenergetics [28,29]. In rodent models, both α5 and PD-E07 mixtures have demonstrated superior effects in promoting mitochondrial biogenesis and offering protection against oxidative stress compared to the BCAAem [30].

There is a scarcity of comparative or randomized controlled intervention studies examining the effects of various amino acid formulations on patients with severe obesity, and the outcomes of existing studies remain unclear [16,31]. Therefore, the objective of the current study was to evaluate the impact of different protein- or amino acid-based supplements on body composition, with a specific focus on MM, as well as muscle strength, metabolic parameters, and inflammatory markers among individuals with severe obesity participating in a multidisciplinary weight loss intervention program.

## 2. Materials and Methods

### 2.1. Study Design

The present study was a 4-week, randomized, controlled trial conducted at the IRCCS Istituto Auxologico Italiano, San Giuseppe Hospital, Piancavallo, Verbania, Italy. The study was registered at Clinicaltrials.gov (NCT05143398) and approved by the Ethical Committee of IRCCS Istituto Auxologico Italiano (approval code #2018_06_28_05). All enrolled patients gave their informed consent to the study participation. The study was conducted according to the Helsinki Declaration.

### 2.2. Participants and Treatments

**Figure 1:**
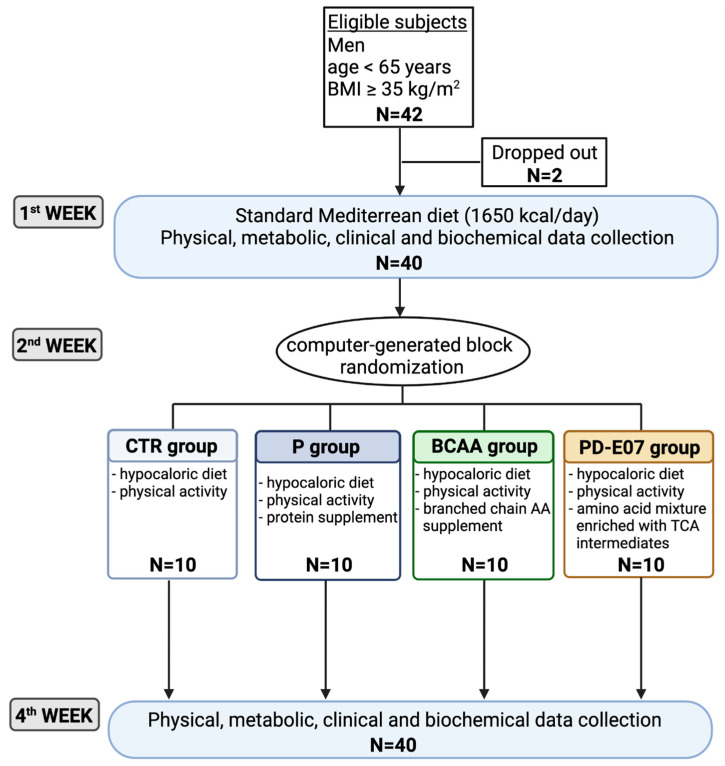
Study design. This study enrolled 42 hospitalized morbidly obese patients; 2 dropped out for personal reasons before randomization. During the first week of hospitalization, patients were subjected to clinical, physical, and biochemical assessments (baseline phase). After an interview with dieticians and psychologists, each patient (N = 40) was subjected to the rehabilitation program (hypocaloric diet and physical activity) and was randomly assigned to a different group of treatment (i.e., CTR, control group, N = 10; P, Protein supplementation group, N = 10; BCAA, Branched Chain Amino Acid supplementation group, N = 10; and PD-E07, Branched Chain Amino Acid and tricarboxylic Acids supplementation group, N = 10 group). At the end of treatment (4 weeks of hospitalization), all patients (N = 40) were subjected to clinical, physical, and biochemical assessments as at baseline (end phase).

We enrolled adult men (18 < age < 65 years) with BMI ≥ 35 kg/m^2^ who were admitted to the hospital for a comprehensive weight loss intervention program that involved metabolic, nutritional, and psychological aspects. Exclusion criteria were previous weight loss interventions within the past year, bariatric surgery, type 2 diabetes, ongoing or recent vitamin, and amino acid supplementation within the last three weeks, hormonal therapy (such as L-thyroxine or testosterone), known muscle atrophies that hinder physical activity (e.g., dystrophies, muscle atrophies, myasthenia gravis), known malignancy, heart failure categorized as New York Heart Association NYHA class IV, end-stage renal disease, liver cirrhosis, tube/percutaneous endoscopic gastrostomy feeding or parenteral nutrition, and patients with no swallowing difficulties or issues with medication intakes. Baseline evaluations were conducted during this period, assessing clinical and nutritional status, energy expenditure, muscle strength and performance, and biochemical and metabolic markers. This week was considered a wash-out period compared to their usual dietary habits. From the second week of hospitalization until discharge, participants were provided with an individualized low-calorie diet plan after consultation with dieticians. Following an interview with the dieticians, a low-calorie diet plan was set up from the second week of hospitalization until discharge. At the end of the baseline evaluation, eligible subjects were randomly assigned to one of the four experimental groups using a computer-generated block randomization, as summarized in Figure 1: (1) diet and physical activity (control group, CTR); (2) diet and physical activity plus protein supplementation (Protifar^®^, Nutricia, Milan, Italy, 2.5 g twice/day corresponding to 4.4 g protein) (P group); (3) diet and physical activity plus BCAA supplementation (Friliver^®^, Dompé, Milan, Italy, 10 g twice/die) (BCAA group); and (4) diet and physical activity plus the PD-E07 formula, an essential amino acid mixture enriched with intermediates of the tricarboxylic acid cycle kindly supplied by Professional Dietetics S.p.A., Milan, Italy (AminoTher PRO^®^, Professional Dietetics, Milan, Italy, 5 g twice/die,) (PD-E07 group). Supplement detailed composition is reported in Supplementary Table S1. After the four-week treatment period, all measurements were repeated, which aligned with the baseline evaluation.

### 2.3. Metabolic-Nutritional-Psychological Rehabilitation Program

The intervention program included individual nutritional education sessions, peer-group psychological support, and supervised physical activity. All patients received a balanced hypocaloric Mediterranean diet with or without protein (P) or amino acid-based supplements (BCAA or PD-E07) (Figure 1). The Mediterranean diet, based on fresh foods, was low in sodium salt (≤3 g/day), and simple sugars (≤10 g/day, essentially fresh fruits derived) were used accordingly to national guidelines for obesity treatment [1,2]. The diet composition was 18–20% proteins (from foods), 27–30% fats (<8% saturated fat), 50–55% carbohydrates (<15% simple sugars), and 30 g of fibers from fresh vegetables. The diet plan was organized into three meals: breakfast (8–9 a.m.), lunch (12 a.m.–1 p.m.), and dinner (6–7 p.m.), with a macronutrient energy distribution of 20%, 40%, and 40%, respectively. All the supplements were administered twice/day at 9 a.m. and 4 p.m. in a glass of water. A nurse monitored the intake of the supplements.

The physical activity program was based on the individual skills of patients for movement, assessed with a visual analogic scale, while taking into consideration possible pain. The motor activity program included one hour of outdoor aerobic activity (walking) and an average of 15–45 min ergometer for five days/week. The endurance training activity was monitored by evaluating each subject’s heart rate and perception of the effort scale (Borg scale CR-10). Physical activity, expressed in the unit of metabolic equivalent (MET), was transformed into kcal. Therefore, the estimated energy expenditure due to physical activity was about 450 kcal/day in all four groups, and the energy expenditure deficit due to diet and physical activity was about −15% of total daily energy requirements.

### 2.4. Anthropometric and Body Composition

During the first seven days of hospitalization, patients carried out the routine diagnostic assessments required to enroll the patients to be included in the intervention (including medical history and blood pressure measurements). Body weight (kg) and body height (m) were measured to the nearest 0.1 kg and 0.5 cm, respectively, using a mechanical column scale (Scale-Tronix, Wheaton, IL, USA) and a stadiometer (Scale-Tronix, Wheaton, IL, USA), and BMI was calculated as body weight/height squared (kg/m^2^). Waist circumference measurements have been made to the nearest 0.1 cm using a tape measure at the uppermost lateral border of the hip crest (ilium). Body composition analysis was carried out with impedance measurements performed in the early morning, after 12-h overnight fasting, using a phase-sensitive, single-frequency bioimpedance analyzer (BIA 101, Akern^®^, Pisa, Italy), which applies an alternating current of 400 microÅ at 50 kHz. Before the measurement, each subject removed clothing and metal jewelry and rested supine for five minutes to equilibrate body fluids. The impedance measurements were made with the patients with a leg opening of approximately 45° compared to the median line of the body and the upper limbs positioned about 30° away from the trunk. After cleaning the skin with alcohol, two Ag/AgCl low-impedance electrodes (Biatrodes, Akern^®^ Srl, Florence, Italy) were placed on the back of the right hand and two electrodes on the corresponding foot at a distance of 5 cm between each other. Before each test session, the device was calibrated using the standard control circuit supplied by the manufacturer with a known impedance (Rz = 380 W and Xc = 47 W, 1% error). The mean coefficient of variation was 1% for within-day and 3% for intra-individual measurements in the steady-state condition. The mean coefficient of variation for the inter-operator variability was 2%. The FFM (kg) was calculated using the population-based prediction model [32,33]. The fat mass (FM) was calculated by the difference between body weight and FFM and expressed as a body weight percentage. We calculated total skeletal muscle mass (MM, kg) using the prediction equation of Janssen et al. [33] and the percentage of MM on body weight.

### 2.5. Energy Expenditure Assessment

Resting energy expenditure (REE) was assessed with indirect computerized calorimetry (Oxycon Pro, VIASYS Healthcare^®^, USA-Yorba Linda, CA, USA) during the morning after overnight fasting in patients who avoided smoking at least 24 h before the evaluation. The REE value (i.e., basal metabolic rate parameter) is necessary to set up the diet plan to induce an energy deficit of ~15% of daily requirements and was calculated using the Weir equation [kcal/d = 1.44 ∗ (3.94 ∗ VO_2_ + 1.11 ∗ VCO_2_)].

### 2.6. Biochemical and Metabolic Marker Measurements

Circulating metabolic parameters, such as glucose, hemoglobin A1c (HbA1c), triglycerides, low-density (LDL-cholesterol) and high-density (HDL-cholesterol) lipoprotein, were measured in fasted patients at baseline and the end of the study (Roche Diagnostics, Mannheim, Germany). Insulin levels were measured by a chemiluminescent assay (Roche Diagnostics). Circulating muscular cytokines, e.g., irisin, amino-terminal of type III procollagen peptide (P3NP), and C-terminal fragment of agrin (CAF), were measured using commercially available ELISA kits (Cat. No. AG-45A-0046YEK-KI01, AdipoGen Lifescience, Cat. No. MBS045955. and Cat. No. MBS7606929 MyBiosource.com, respectively) and following manufacturer instructions.

### 2.7. Muscle Strength and Performance

We measured the muscle strength via the handgrip test using a dynamometer (JAMAR^®^ isometric dynamometer, Cedarburg, WI, USA, on both sides, dominant or not). Three measurements were taken for both hands, and mean values were calculated for each hand [34]. Individual global performance was evaluated using the distance in meter walk in 6 min (6 min walking test) [35,36].

### 2.8. Statistical Analysis

The sample size for this pilot study was determined based on the guideline proposed by Julious SA et al. [37]. Continuous variables were presented as mean and standard deviation, assuming normal distribution when applicable. The Shapiro–Wilks test was performed to investigate the Normal distribution of continuous variables. ANOVA models (or Kruskal–Wallis tests) were performed to test absolute differences among the four groups of interest of parameter measured at baseline and at follow-up as well as absolute and percentage differences of changes between follow-up and baseline (delta). Finally, a repeated measure model (considering an unstructured as variance–covariance matrix) was implemented to evaluate the differences of MM (kg) and MM (%) between the control group versus each treatment group. The model included as covariates: (i) group (CTR, P, BCAA, and PD-E07), (ii) time (baseline and end-of-study), and (iii) interaction between group and time. Baseline variables statistically different among groups were included in the model. To address the issue of multiple comparisons, a false discovery rate (FDR) adjustment was applied. The analyses were conducted using SAS software (Version 9.4; SAS Institute, Cary, NC, USA). A significance level of less than 0.05 for two-tailed *p*-values was considered statistically significant.

## 3. Results

### 3.1. Effects of Nutritional Supplements on Body Weight and Body Composition

The study involved 40 male in-patients with morbid obesity, with 10 patients in each study group (Table 1). All patients successfully completed the intervention. Following the treatment, we observed significant improvements in waist circumference, blood pressure, glucose levels, and lipid metabolism parameters across the entire patient group (Table 1).

**Figure 2:**
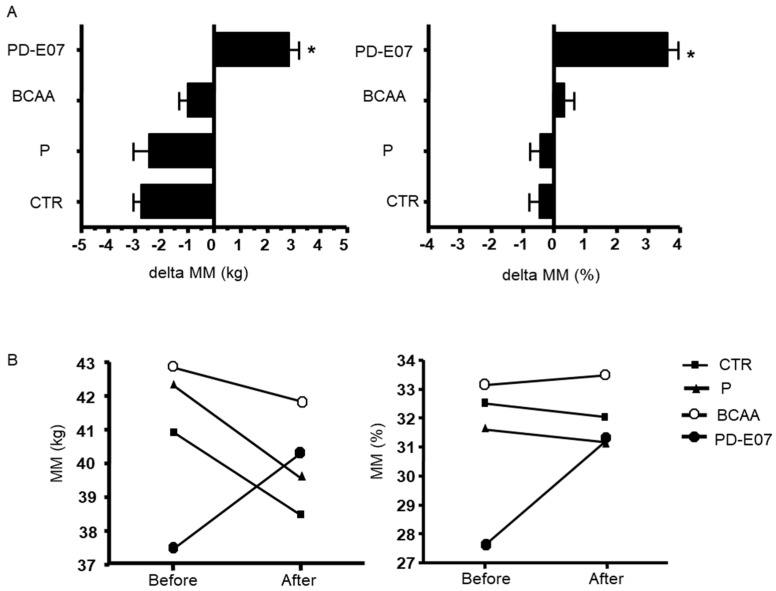
Panel (**A**), Delta of muscle mass, MM (expressed as kg on the left panel and % of the weight on the right panel) in the four groups of intervention (CTR, control group; P, Protein supplementation group; BCAA, Branched Chain Amino Acid supplementation group; and PD−E07, Branched Chain Amino Acid and tri−carboxylic Acids supplementation group); Panel (**B**), MM trends (as kg on the left panel and % of weight in the right panel) in the four groups of intervention (see above). * *p* < 0.05 between CTR and PD−E07.

Upon randomization at baseline, there were no significant differences among the intervention groups regarding the considered clinical parameters, except for MM expressed as a percentage of body weight (*p* < 0.05). The kg of MM did not show significant differences among the four groups at baseline (Supplementary Table S2). Supplementary Table S2 presents the baseline clinical characteristics stratified by treatment group. The median age in the control group was 56 years, whereas it was 51.5 years in both the BCAA and PD-E07 groups. The P group had the youngest median age of 49 (*p* = 0.0735). The patients, after randomization, exhibited similar degrees of obesity (severe obesity, class III, BMI > 40 kg/m^2^) and comparable waist measurements. At the end of the intervention, no differences in body weight or most parameters considered between the four groups of treatments were observed (detailed in Supplementary Table S3). When analyzing the delta values, a significant increase in MM in the PD-E07 intervention group [MM (kg): 2.84 ± 3.57; MM (%): 3.63 ± 3.14] compared to the CTR group [MM (kg): −2.46 ± 3.04; MM (%): −0.47 ± 2.28] (Figure 2) was observed (*p* = 0.045 and *p* = 0.023 respectively). The MM values of the P [MM (kg): −2.75 ± 5.98, *p*= 0.734; MM (%): −0.44 ± 4.02, *p*= 0.990] and BCAA [MM (kg): −1 ± 3.3, *p* = 0.734; MM (%): 0.34 ± 2.85, *p* = 0.956] did not exhibit a statistically significant difference when compared to the CTR group (Figure 2).

### 3.2. Effect of Nutritional Supplements on Glucose Homeostasis and Lipid Metabolism

After rehabilitation, fasting glucose, insulin, and Hb1Ac levels were improved in all intervention groups (Table 2). No significant differences were observed when comparing the CTR and the supplemented groups. In addition, all groups showed significant reductions in circulating LDL-cholesterol and TG levels.

### 3.3. Effects of Nutritional Supplements on Physical Performance and Muscle Health

The 6-min walking test and the distance walked increased across all study groups, with no significant differences between them (Table 2). Among all study groups, there was no difference in muscle strength measured by handgrip strength. Additionally, no significant changes were observed in the plasma levels of specific myokines in any of the treatment groups (Table 2).

## 4. Discussion

This pilot comparative study demonstrates that amino acid-based supplementation can potentially prevent the loss of muscle mass (MM) typically associated with weight loss in patients with severe obesity. However, this positive effect observed on MM was not accompanied by changes in physical performance, as assessed by the 6-min walking test, or muscle strength, measured by handgrip, as these outcomes were comparable across all studied groups. Furthermore, no significant differences were observed in glucose and lipid metabolism and in the levels of specific myokines.

The hypocaloric diet combined with regular physical activity has already been shown to attenuate the reduction in MM associated with diet-induced weight loss [38,39]. However, controversial results have been reported on the body composition of individuals with obesity due to various interventions: e.g., type of exercise, aerobic vs. resistance training, and high protein intake vs. low protein intake (i.e., less than the recommended daily intake of 0.8 g per day). One of these studies found that high protein intake (>1.5 g/kg) in obese subjects during weight loss [16] maintained lean body mass and MM but did not improve muscle strength and metabolic homeostasis [5]. Another paper reported that high consumption of dietary proteins, particularly whey proteins (i.e., high-quality proteins), promoted muscle protein synthesis during diet-induced weight loss with or without metabolic effects [38]. These beneficial effects of whey proteins have been attributed to their high content of EAA and BCAA, particularly leucine, which strongly stimulates muscle protein synthesis. Accordingly, several studies reported positive effects of EAA supplementation on muscle protein maintenance [39,40]. Our results, obtained in a short intervention, confirm that the standard rehabilitation program produced a body weight loss of 5%. Although several reports showed that supplementation of BCAA mixtures increased MM and muscle strength [16,41,42], we did not observe comparable effects. We observed a significant increase in MM (kg and percentage) in the PD-E07 intervention group. Recently, in a comparative study (BCCA supplementation vs. high protein intake), a significant improvement in calf muscle volume was registered in the BCAA-supplemented group after weight loss, and this observation was justified by a reduction in fat infiltration in muscle cells documented by distal muscle MRI [43].

The BCAAs are essential precursors of the intermediates of the TCA cycle via acetyl-CoA and succinyl-CoA synthesis and, thus, are involved in energy production. These metabolic effects may be relevant to explain the beneficial effects of protein consumption on muscle strength and physical function during weight loss, although the results are inconclusive. In particular, some studies showed an improvement in the 6 min walking test in the elderly population as an index of cardiovascular performance after amino acid supplementation [44]. No significant differences in functional tests (6MWT or HG) were observed in our studied patients for either amino acid formulation versus controls or protein supplementation. Longer follow-ups are probably needed.

Based on previous data [43], glucose and lipid metabolism did not differ significantly between the groups studied. These results consistently show a significant improvement in insulin sensitivity in patients with metabolic syndrome [45] after weight loss with associated diet and exercise. In contrast, animal protein consumption promotes insulin resistance [46], possibly through high BCAA content, although the exact mechanisms are unclear [47]. Accordingly, increased circulating levels of BCAA or their breakdown products (i.e., branched-chain α-keto acids) are associated with insulin resistance [48,49]. To better understand the mechanism underlying the effect of dietary supplements, we examined safety and efficacy markers of skeletal muscle health. These markers included the C-terminal fragment of agrin (CAF), a proteoglycan of the glomerular and tubular basement membrane that appears to be a more reliable indicator of muscle wasting than deterioration in muscle strength in healthy older adults [50]. We also studied irisin, a hormone produced by skeletal muscle that controls various processes such as hepatic glucose and lipid metabolism, brain function, and bone remodeling [51], along with amino-terminal levels of procollagen type III (P3NP) [52]. None of these biochemical markers of muscle metabolism appear to explain our findings.

Although this pilot comparative study provides evidence suggesting that amino acid-based supplementation has the potential to prevent muscle mass (MM) loss during weight loss, it is important to acknowledge the limitations of the current study. Firstly, due to its pilot nature and small sample size, it is crucial to replicate the intervention in a larger cohort of patients with obesity, including those with and without sarcopenia, to evaluate the supplementation benefits across different phenotypes. Secondly, the study focused solely on male participants, and therefore, the effects of supplementation on female subjects remain unknown. Lastly, to comprehensively assess efficacy, it is necessary to investigate the impact of higher doses of dietary supplements over an extended time period of supplementation.

## 5. Conclusions

Our results demonstrate that incorporating amino acids supplementation, particularly formulations rich in essential and branched-chain amino acids, may benefit individuals undergoing weight loss programs to preserve muscle mass. However, it is crucial to consider that other factors, such as overall dietary protein intake, type of exercise, and individual variability, may influence the outcomes. Therefore, personalized approaches tailored to individual needs and goals should be considered when implementing amino acids supplementation in weight loss interventions. Overall, this study contributes to the growing body of knowledge on the potential role of amino acids in weight loss programs. Future research endeavors in using amino acids in weight loss programs should aim to investigate the optimal dosage, duration, and timing of amino acids supplementation.

## Figures and Tables

**Table 1:** Anthropometric and biochemical clinical characteristics of the studied cohort. Data are expressed as mean ± standard deviation (SD) before and 4 weeks after the intervention.

ALL (N = 40)	Before	After		*p*-Value *
Age (years)	52.55 ± 5.06	-	-	
Weight (kg)	133.38 ± 21.39	126.24 ± 19.17		Ns
BMI (Kg/m^2^)	44.45 ± 6.54	42.04 ± 5.83		Ns
Waist (cm)	133.28 ± 12.47	125.55 ± 10.99		0.005
Sys P (mmHg)	147.31 ± 18.53	124.62 ± 11.40		<0.0001
Diast P (mmHg)	87.44 ± 8.69	79.36 ± 8.18		<0.0001
Heart Rate (bpm)	80.74 ± 10.71	75.79 ± 10.62		0.05
FM (kg)	56.71 ± 14.23	52.70 ± 13.62		Ns
FM (%)	42.31 ± 4.41	41.07 ± 4.98		Ns
FFM (kg)	75.61 ± 11.02	74.38 ± 9.59		Ns
FFM (%)	57.57 ± 4.51	58.94 ± 4.98		Ns
MM (kg)	40.89 ± 5.99	40.04 ± 4.64		Ns
MM (%)	31.24 ± 4.52	32.00 ± 4.49		Ns
REE (Kcal/die)	2105.64 ± 257.73	2022.44 ± 256.45		Ns
Glucose (mg/dL)	97.70 ± 10.18	91.03 ± 8.01		0.002
Insulin (mU/L)	18.79 ± 5.91	17.36 ± 6.96		Ns
HbA1c (%)	5.79 ± 0.39	5.53 ± 0.48		0.01
HDL (mg/dL)	38.65 ± 6.72	35.30 ± 6.05		0.023
LDL (mg/dL)	132.28 ± 36.39	113.40 ± 29.98		0.01
TG (mg/dL)	159.80 ± 42.42	134.33 ± 37.63		0.006
6MWT (meters)	477.06 ± 132.66	531.00 ± 81.68		0.04
HGS (right arm)	44.70 ± 9.14	46.41 ± 9.77		Ns
HGS (left arm)	41.76 ± 8.50	43.42 ± 9.23		Ns

Abbreviations: BMI: Body Mass Index; Sys P: Systolic Blood pressure; Diast P: Diastolic Blood Pressure; FM: Fat Mass; FFM: Fat-free Mass; MM: Muscular Mass; REE: Resting Energy expenditure; HbA1c: Glycated hemoglobin; HDL: high-density lipoproteins; LDL: low-density lipoproteins; TG: triglycerides; 6MWT: 6-min walking test; HGS: Handgrip Strength. * Wilcoxon signed-rank test.

**Table 2:** Delta of anthropometric and biochemical clinical characteristics of four intervention groups (CTR, control group, N = 10; P, Protein supplementation group, N = 10; BCAA, Branched Chain Amino Acid supplementation group, N = 10; and PD-E07, Branched Chain Amino Acid and tri-carboxylic Acids supplementation group, N = 10). Data are expressed as mean ± standard deviation (SD) of delta difference after/before 4 weeks of intervention.

	CTR	P	AA	PD-E07	*p*-Value ^†^
Weight (kg)	−6.77 (2.5)	−8.34 (4.39)	−6.12 (1.11)	−7.3 (3.03)	0.4129
Weight (%)	−5.23 (1.43)	−5.86 (2.19)	−4.67 (0.63)	−5.16 (1.5)	0.4016
BMI (kg/m^2^)	−2.24 (0.79)	−2.72 (1.38)	−2.1 (0.36)	−2.55 (1.07)	0.4803
BMI (%)	−5.24 (1.44)	−5.84 (2.22)	−4.77 (0.74)	−5.35 (1.51)	0.5118
Waist (cm)	−6.3 (3.43)	−11.8 (7.25)	−4.4 (5.15)	−8.4 (7.86)	0.0648
Waist (%)	−4.67 (2.34)	−8.63 (4.93)	−3.23 (3.77)	−6.08 (5.42)	0.0491
Sys P (mmHg)	−22.5 (19.76)	−12 (50.67)	−13.89 (13.18)	−24.5 (23.86)	0.7546
Diast P (mmHg)	−12 (11.83)	2 (28.89)	−7.78 (13.25)	−4.5 (8.96)	0.3561
Heart rate (bpm)	−4.5 (15.63)	3.4 (28.38)	−2.56 (13.53)	−7.8 (14.97)	0.6200
FM (kg)	−2.62 (4.08)	−1.87 (5.71)	−5.6 (2.19)	−5.94 (5.85)	0.1425
FM (%)	−0.14 (3.16)	0 (4.68)	−2.52 (1.62)	−2.3 (4.09)	0.2494
FFM (kg)	−3.03 (4.03)	−4.13 (7.75)	2.21 (4.73)	0.04 (4.89)	0.0582
FFM (%)	0.12 (3.15)	0.03 (4.68)	3 (1.29)	2.3 (4.09)	0.1600
MM (kg)	−2.46 (3.04)	−2.75 (5.98)	−1 (3.3)	2.84 (3.57)	0.0172
MM (%)	−0.47 (2.28)	−0.44 (4.02)	0.34 (2.85)	3.63 (3.14)	0.0172
REE (kCal/die)	−116.75 (122.41)	−41.43 (78.13)	−123.7 (148.5)	−167.43 (158.24)	0.3651
Glucose (mg/dL)	−9.5 (14.19)	−4.4 (7.32)	−2.7 (7.09)	−10.1 (10.41)	0.2856
Insulin (mU/L)	−2.96 (9.55)	1.17 (7.53)	−3.23 (4.2)	−0.89 (5.15)	0.4695
HDL (mg/dL)	−2.2 (4.98)	−3.9 (6.38)	−4.2 (3.74)	−3.1 (4.79)	0.8156
LDL (mg/dL)	−1.4 (34.63)	−18.2 (25.13)	−36.1 (34)	−19.8 (12)	0.0694
TG (mg/dL)	−27 (20.31)	−13 (40.38)	−34.5 (27.37)	−27.4 (30.23)	0.4633
HbA1c (%)	−0.33 (0.21)	−0.34 (0.6)	−0.14 (0.21)	−0.21 (0.2)	0.5240
6MWT (meters)	104.6 (197.4)	54.4 (132.44)	43.63 (37.01)	59.33 (77.2)	0.7532
HGS (right arm, Kg)	1.06 (2.45)	3.63 (5.47)	0.52 (4.4)	1.62 (6.93)	0.5400
HGS (left arm, Kg)	0.75 (1.91)	3.39 (5.21)	1.79 (4.76)	0.59 (7.2)	0.5976
CAF (μg/mL)	3.3 (21.91)	−5.52 (20.97)	5.55 (12.83)	58.92 (177.52)	0.4521
Irisin (μg/mL)	−0.86 (3.32)	8.79 (14.78)	3.92 (14.06)	0.64 (3.92)	0.2876
P3NP (μg/mL)	4.53 (13.92)	−1.53 (14.66)	−4.08 (5.31)	−0.66 (5.24)	0.4188

Abbreviations: BMI: Body Mass Index; Sys P: Systolic Blood pressure; Diast P: Diastolic Blood Pressure; FM: Fat Mass; FFM: Fat-free Mass; MM: Muscular Mass; REE: Resting Energy expenditure; HbA1c: Glycated hemoglobin; HDL: high-density lipoproteins; LDL: low-density lipoproteins; TG: triglycerides; 6MWT: 6-min walking test; HGS: Handgrip Strength; CAF: C-terminal agrin fragment; P3NP: procollagen type III N-terminal peptide. ^†^ ANOVA model.

## Data Availability

The datasets generated and/or analyzed during the current study are not publicly available but are available from the corresponding author upon reasonable request.

## Supplementary Materials

The following supporting information can be downloaded at: https://www.mdpi.com/article/10.3390/jcm12134257/s1, Table S1: Nutritional supplement used in the intervention groups…; Table S2: Anthropometric and clinical parameters measured after randomization in the four study groups…; Table S3: Anthropometric and clinical parameters in the 4 study groups…. after 4 weeks of intervention.
