# Supplementary material for: Comparison of Protein- or Amino Acid-Based Supplements in the Rehabilitation of Men with Severe Obesity: A Randomized Controlled Pilot Study
Source: J Clin Med. 2023 Jun 25;12(13):4257. doi: 10.3390/jcm12134257 (PMC10342837; doi:10.3390/jcm12134257)
Supplement: Supplementary file 1 [file jcm-12-04257-s001.zip › jcm-2307683-supplementary.pdf]

Supplementary Table S1

Nutritional supplement used in the intervention groups (P, proteins, BCAA, Branched Chain Amino Acids, PD-E07, Branched Chain Amino Acids and tricarboxylic acids). The composition and commercial name are indicated.

	**P**<br>*Protifar®*	**BCAA**<br>*Friliver®*	**PD-E07**<br>*Amino-Ther® PRO*
	**g/100 g**	**g/100 g**	**g/100 g**
**Macronutrients (g)**			
Protein	87.2	-	-
Carbohydrate	1.2	66.9	36.8
Polyalcohol	-	10	-
Fat	1.6	-	-
Saturated	1.2	-	-
Unsaturated	0.4	-	-
**Minerals (mg)**			
Na	110	-	-
K	140	-	-
Ca^{++}	1350	-	-
P	700	-	-
Mg	≤ 20	-	-
Cl	80	-	-
**Amino acids. vitamins and TCA precursors (g)**			
Leucine	-	12	23.46
Isoleucine	-	6	17.59
Valine	-	6	13.68
Lysine	-	-	11.73
Threonine	-	-	11.73
Histidine	-	-	2.93
Cysteine/cystine	-	-	2.93
Phenylalanine	-	-	1.95
Methionine	-	-	0.98
Tryptophan	-	-	0.98
Vit B6	-	-	0.02
Vit B1	-	-	0.01
Citric acid	-	-	8.00
Succinic acid	-	-	2.00
Malic acid	-	-	2.00
**Calories (%)**			
Protein or amino acid	95	26	68
Carbohydrate	1	74	32
Fat	4	0	0
**Total energy (kcal)**	368.0	260.0	402.0

Supplementary Table S2 – Anthropometric and clinical parameters measured after randomization in the four study groups (CTR, control group; P, Protein supplementation group; BCAA, Branched Chain Amino Acid supplementation group; and PD-E07, Branched Chain Amino Acid and tri-carboxylic Acids supplementation group) at baseline (before the 4 weeks intervention). Data are expressed as mean (± standard deviation, SD).

	CTR (N=10)	P (N=10)	AA (N=10)	PD-E07 (N=10)	P-value†
Age (years)	55.5(4.22)	49.7(3.8)	52(4.29)	53(6.63)	0.0803
Weight (kg)	127.48(19.72)	137.33(26.49)	130.65(13.39)	138.04(25.99)	0.6544
BMI (Kg/m^2)	42.09(4.98)	45.55(7.15)	44.09(3.16)	46.06(9.67)	0.5543
Waist (cm)	131.5(14.83)	134.3(12.23)	132.5(8.75)	134.8(15.4)	0.9360
Sys P (mmHg)	142.5(12.75)	146.67(21.65)	147(15.67)	153(24.52)	0.6787
Diast P (mmHg)	88.5(7.84)	86.11(7.82)	89.5(10.12)	85.5(9.85)	0.7252
Heart Rate (n/min)	81.1(12.9)	81.44(10.6)	81(10.82)	79.5(10.54)	0.9817
FM (kg)	52.61(12.1)	55.29(12.66)	55.99(7.21)	62.93(21.84)	0.4382
FM (%)	41.06(3.1)	40.44(2.43)	42.91(3.31)	44.82(6.86)	0.1143
FFM (kg)	74.43(8.31)	80.93(14.35)	72.45(12.03)	74.61(8.67)	0.3635
FFM (%)	58.94(3.1)	59.56(2.43)	56.61(3.71)	55.18(6.86)	0.1077
MM (Kg)	40.93(4.52)	42.32(8.07)	42.83(4.57)	37.46(5.79)	0.1898
MM (%)	32.52(3.82)	31.62(5.75)	33.16(2.77)	27.64(3.89)	0.0254
REE (Kcal/die)	2126.38(229.4)	2036.6(262.7)	2077.4(243.1)	2206.5(322.9)	0.5793
Glucose (mg/dl)	96.2(14.18)	97.4(9.32)	95.6(7.43)	101.6(9.64)	0.5752
Insulin (mU/L)	20.9(7.08)	16.56(5.06)	18.38(5.94)	19.98(6.09)	0.4495
HDL (mg/dl)	36.7(6.09)	37.7(9.07)	39.9(5.82)	40.3(6.13)	0.6015
LDL (mg/dl)	116.2(37.14)	129.9(31.8)	157.6(32.47)	125.4(37.16)	0.0648
TG (mg/dl)	157.3(46.52)	154.2(47.23)	168.1(51.52)	159.6(28.8)	0.9091
HBA1c (%)	5.88(0.47)	5.81(0.44)	5.69(0.27)	5.77(0.41)	0.7622
6MWT (meters)	504.22(72.25)	509.67(41.3)	466.11(184.3)	428.22(182.93)	0.5619
HGS (right arm, Kg)	41.72(4.78)	44.02(12.76)	45.5(9.49)	47.87(8.57)	0.5453
HGS (left arm, Kg)	40.21(4.95)	40.46(11.19)	40.73(8.39)	46.07(8.81)	0.4135
CAF (ug/ml)	95.05(98.34)	52.7(36.27)	26.62(21.65)	292.42(541)	0.2153
Irisin (ug/ml)	8.05(7.18)	18.8(15.55)	11.83(7.81)	8.64(4.25)	0.1236
P3NP (ug/ml)	17.59(22.11)	26.51(26.5)	19.62(14.81)	25.86(24.28)	0.7860

Abbreviations: BMI: Body Mass Index; Sys P: Systolic Blood pressure; Diast P: Diastolic Blood Pressure; FM: Fat Mass; FFM: Fat free Mass; MM: Muscular Mass; REE: Resting Energy expenditure; HbA1c: Glycated haemoglobin; HDL: high density lipoproteins; LDL: low density lipoproteins; TG: triglycerides; 6MWT: six minutes walking test; HGS: Handgrip Strength; CAF: C-terminal agrin fragment; P3NP: procollagen type III N-terminal peptide † ANOVA model

Supplementary Table S3 – Anthropometric and clinical parameters in the 4 study groups (CTR, control group; P, Protein supplementation group; BCAA, Branched Chain Amino Acid supplementation group; and PD-E07, Branched Chain Amino Acid and tri-carboxylic Acids supplementation group) after the 4 weeks intervention. Data are expressed as mean and (standard deviation).

	**CTR (N=10)**	**P (N=10)**	**AA (N=10)**	**PD to E07 (N=10)**	**P-value †**
Weight (kg)	120.71(17.81)	128.99(23.17)	124.53(12.68)	130.74(23.47)	0.6697
BMI (Kg/m^2)	39.84(4.36)	42.83(6.21)	41.99(3.03)	43.51(8.7)	0.5515
Waist (cm)	125.2(12.8)	122.5(10.33)	128.1(7.82)	126.4(13.66)	0.7340
Sys P (mmHg)	120(11.3)	120(11.55)	130.56(9.5)	128.5(11.07)	0.0755
Diast P (mmHg)	76.5(9.44)	79.5(5.99)	80.56(8.08)	81(9.66)	0.6378
Heart Rate (n/min)	76.6(10.96)	76.7(8.84)	78.44(8.37)	71.7(14.17)	0.5642
FM (kg)	49.99(10.29)	53.42(15.95)	50.39(5.93)	56.99(19.86)	0.6623
FM (%)	40.92(3.35)	40.44(5.65)	40.39(3.64)	42.52(7.11)	0.7735
FFM (kg)	71.4(8.59)	76.8(10.75)	74.66(9.73)	74.65(10.4)	0.6806
FFM (%)	59.06(3.31)	59.59(5.66)	59.61(3.64)	57.48(7.11)	0.7702
MM (Kg)	38.47(3.87)	39.57(4.69)	41.83(5.13)	40.3(5.09)	0.4553
MM (%)	32.05(3.98)	31.18(5.97)	33.5(3.11)	31.27(4.94)	0.6589
REE (Kcal/die)	2009.63(238.5)	2045.86(280.5)	1953.7(261.4)	2111.86(291.8)	0.6808
Glucose (mg/dl)	86.7(3.97)	93(12.43)	92.9(8.1)	91.5(4.48)	0.2667
Insulin (mU/L)	17.94(7.37)	17.73(8.19)	15.15(6.37)	19.09(6.74)	0.6912
HDL (mg/dl)	34.5(5.52)	33.8(8.08)	35.7(3.68)	37.2(6.76)	0.6367
LDL (mg/dl)	114.8(18.75)	111.7(33.49)	121.5(34.96)	105.6(33.95)	0.7154
TG (mg/dl)	130.3(44.35)	141.2(30.73)	133.6(50.93)	132.2(26.67)	0.9310
HbA1c (%)	5.55(0.45)	5.47(0.77)	5.55(0.29)	5.56(0.35)	0.9753
6MWT (meters)	558.4(67.96)	513.1(46.75)	563.89(45.98)	487.56(131.3)	0.1380
HGS (right arm, Kg)	42.78(4.93)	47.65(10.68)	46.02(10.12)	49.49(12.87)	0.5122
HGS (left arm, Kg)	40.96(4.15)	43.85(11.37)	42.52(8.94)	46.66(11.84)	0.6141
CAF (ug/ml)	98.35(102.48)	47.18(32.58)	36.73(29.71)	351.34(698.49)	0.2256
Irisin (ug/ml)	7.18(5.08)	27.59(28.75)	15.75(18.07)	9.27(6.52)	0.1094
P3NP (ug/ml)	22.12(21.49)	24.98(23.3)	15.54(13.33)	25.2(22.52)	0.7041

Abbreviations: BMI: Body Mass Index; Sys P: Systolic Blood pressure; Diast P: Diastolic Blood Pressure; FM: Fat Mass; FFM: Fat free Mass; MM: Muscular Mass; REE: Resting Energy expenditure; HbA1c: Glycated haemoglobin; HDL: high density lipoproteins; LDL: low density lipoproteins; TG: triglycerides; 6MWT: six minutes walking test; HGS: Handgrip Strength; CAF: C-terminal agrin fragment; P3NP: procollagen type III N-terminal peptide.
† ANOVA model
